# Integrate-and-fire-type models of the lateral superior olive

**DOI:** 10.1371/journal.pone.0304832

**Published:** 2024-06-20

**Authors:** Go Ashida, Tiezhi Wang, Jutta Kretzberg

**Affiliations:** 1 Faculty 6, Department of Neuroscience, Carl von Ossietzky Universität Oldenburg, Oldenburg, Germany; 2 Cluster of Excellence "Hearing4all", Carl von Ossietzky Universität Oldenburg, Oldenburg, Germany; 3 Faculty 6, Department of Health Services Research, Carl von Ossietzky Universität Oldenburg, Oldenburg, Germany; 4 Research Center Neurosensory Science, Carl von Ossietzky Universität Oldenburg, Oldenburg, Germany; University of Miami, UNITED STATES

## Abstract

Neurons of the lateral superior olive (LSO) in the auditory brainstem play a fundamental role in binaural sound localization. Previous theoretical studies developed various types of neuronal models to study the physiological functions of the LSO. These models were usually tuned to a small set of physiological data with specific aims in mind. Therefore, it is unclear whether and how they can be related to each other, how widely applicable they are, and which model is suitable for what purposes. In this study, we address these questions for six different single-compartment integrate-and-fire (IF) type LSO models. The models are divided into two groups depending on their subthreshold responses: passive (linear) models with only the leak conductance and active (nonlinear) models with an additional low-voltage-activated potassium conductance that is prevalent among the auditory system. Each of these two groups is further subdivided into three subtypes according to the spike generation mechanism: one with simple threshold-crossing detection and voltage reset, one with threshold-crossing detection plus a current to mimic spike shapes, and one with a depolarizing exponential current for spiking. In our simulations, all six models were driven by identical synaptic inputs and calibrated with common criteria for binaural tuning. The resulting spike rates of the passive models were higher for intensive inputs and lower for temporally structured inputs than those of the active models, confirming the active function of the potassium current. Within each passive or active group, the simulated responses resembled each other, regardless of the spike generation types. These results, in combination with the analysis of computational costs, indicate that an active IF model is more suitable than a passive model for accurately reproducing temporal coding of LSO. The simulation of realistic spike shapes with an extended spiking mechanism added relatively small computational costs.

## Introduction

Computational modeling plays a pivotal role in many fields of natural science, including neurobiology. Neuronal models in auditory neuroscience have revealed, for example, how our brain processes and combines signals from the two ears to form the perception of an acoustic image. One of the primary stations for such binaural information processing is the lateral superior olive (LSO), which is located in the auditory brainstem in the mammalian nervous system [1–3]. As reviewed previously [4–6], a number of different models have been developed to simulate the physiological functions of LSO neurons. These models, however, were usually tuned with a small set of empirical data to meet the specific aims of each modeling study. Therefore, it is generally unclear how comparable these different models are to each other and how applicable they are to a wider range of questions, making it hard to re-use models in other contexts. In our previous study [7], we compared seven types of single-compartment LSO neuron models and found that their simulated physiological responses to binaural stimuli largely resembled each other if they were calibrated with common criteria. Based on these results, we concluded that a user can readily choose an LSO model according to their intended purposes, and that a model with intermediate complexity may serve as a good starting point for general use [7], consistent with general advice to select a medium-sized model [8].

**Fig 1:**
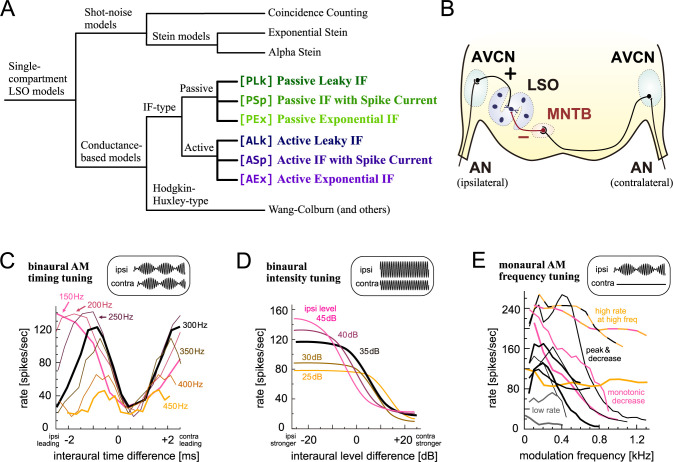
LSO models and relevant physiological response properties. **A:** Interrelations of the LSO models. The six IF-type models examined in this study are color-coded consistently in all figures of this paper. Other single-compartment models shown in black were studied previously [7]. Corresponding equations are given in Materials and Methods (Eqs 1–7). **B:** Schematic drawing of the LSO circuit. Excitatory inputs are shown in black; inhibitory inputs are shown in red. See text for abbreviations. **C:** Spike rate changes of a cat LSO neuron driven by AM tones with varied ITDs. Different lines correspond to different modulation frequencies. **D:** Spike rate changes of a cat LSO neuron driven by binaural unmodulated tones with varied ILDs. Different colors correspond to different ipsilateral sound levels. **E:** Spike rate changes of LSO neurons driven by monaural AM tones with varied modulation frequencies. Different lines show different units and different colors indicate different response types. Panels B-E are adapted from [7] with color modifications; original data in cats were collected by [18] for C and E and by [19] for D.

Among the LSO models we studied before [7], integrate-and-fire (IF) type neuron models have intermediate complexity. Their spike-generating mechanisms are simple threshold-crossing detectors [9], but they still have a membrane potential that is compatible with conductance-based synaptic input models and that can be compared to physiological measurements. IF-type models have been used to simulate the physiological functions and dysfunctions of LSO in binaural processing [10–13]. In the last two decades, a variety of nonlinear IF models have been developed and used [14–16]. Among them, the exponential integrate-and-fire (EIF) model and its variations have been successfully used in many applications [15,17]. We formerly predicted that an EIF-based LSO model would show similar responses to other IF models that we already examined in [7]. In the present study, we aim to test this prediction with the same set of input stimuli and response criteria as used previously. In addition, we expand our list of IF-type LSO models to cover possible combinations of "add-ons". Namely, we start with the passive leaky IF model and make modifications to obtain six different models (Fig 1A) (see the first section of Results and Discussion for their general introduction). Our goals are to examine how similarly or differently these models respond to the same set of stimuli and how much computational cost is added by the modifications of each model.

The following anatomical and physiological properties of LSO comprise the bases of our modeling (see, e.g., [2,3,20,21], for more complete reviews). Excitatory inputs from spherical bushy cells (SBCs) in the ipsilateral anteroventral cochlear nucleus (AVCN), which are innervated by auditory nerve fibers, drive LSO neurons. In addition, inhibitory inputs from the medial nucleus of the trapezoid body (MNTB), which are innervated by globular bushy cells (GBCs) in the contralateral AVCN, suppress the LSO (Fig 1B). This binaural excitatory-inhibitory interaction is the basis of "anticoincidence detection" in LSO neurons [22–26], leading to their sensitivity to interaural time and level differences (ITDs/ILDs). The output spike rate of an LSO neuron varies periodically with the ITD of the envelope of amplitude-modulated (AM) sounds (Fig 1C), and changes monotonically with the ILD of pure tones (Fig 1D). The sensitivity of LSO neurons to these binaural cues is the neuronal basis of binaural sound localization [2,3,20,21]. Furthermore, when stimulated by monaural AM tones, an LSO neuron varies its spike rate with different modulation frequencies, often showing a mild peak at around 100–400 Hz and gradual decay at higher frequencies (Fig 1E). Our modeling aims to replicate these monaural and binaural tuning properties.

## Results and discussion

### IF-type LSO models to compare

**Fig 2:**
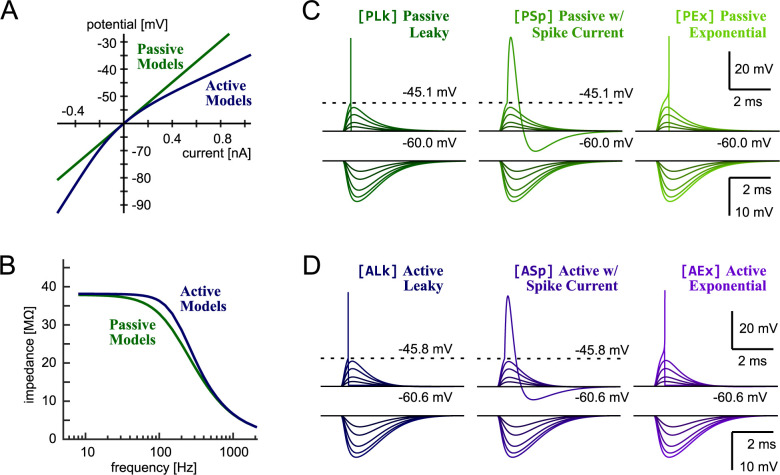
Sub- and suprathreshold model responses. **A:** Current-voltage relationship of the passive (green) and active (blue) LSO models. The spike generation mechanisms of all models were disabled for this panel. **B:** Membrane impedance of the passive (green) and active (blue) models. In A and B, the subtypes of active and passive models were not distinguished, because the subthreshold membrane responses were almost identical regardless of the spike-generation mechanisms. **C:** Responses of passive IF models to excitatory (upper panels) and inhibitory (lower panels) synaptic inputs, both of which were modeled with an alpha function (see Methods). **D:** Responses of active IF models to excitatory and inhibitory synaptic inputs. In each subpanel of C and D, simulated voltage traces for 1, 2, 4, 6, and 8 synchronized synaptic inputs are shown; solid and dotted horizontal lines indicate the resting and threshold voltage levels, respectively. The voltage upswing of the leaky models (PLk and ALk) were added manually to show spike timings, while the spike shapes of the other models resulted from their intrinsic features.

The LSO comprises multiple types of neurons that can be anatomically and physiologically classified [21,27]. Here we simulate the activity of sustained-spiking LSO neurons, as they have been most extensively investigated in vivo [1,20]. The six IF models in this study all shared the fundamental equations (Eq 1 in "Integrate-and-fire models of LSO" of Materials and Methods). They were subdivided into two groups (Fig 1A), according to their subthreshold responses: namely, passive IF and active IF models. The passive model (shown in green in all figures) was based on an RC circuit and its current-voltage (I-V) relationship was linear, while the active models (shown in blue) had an additional low-voltage-activated potassium (KLVA) conductance that led to a nonlinear I-V curve (Fig 2A). KLVA channels are expressed in many different neurons in the auditory system including LSO, and underlie precise temporal coding [28,29]. Both passive and active models acted as low-pass filters, but the active models had higher impedances than the passive models at 40–400 Hz due to the activation of KLVA channels in the subthreshold regime around -60 mV (Fig 2B).

The group of passive models was further divided into three different models (Figs 1A and 2C), according to their spike-generation mechanisms. In the most basic type, called the passive leaky IF (PLk) model, a spike was counted when the simulated membrane potential reaches the threshold, which was followed by an immediate reset of the voltage to the resting state (Fig 2C, left). A possible modification of the model can be achieved by adding a spike-mimicking current [30,31]. In the "passive IF with spike current" (PSp) model, a crossing of the threshold triggered a spike-like response with an up- and down-swing of the voltage (Fig 2C, middle). Another modification we considered was to replace the detection of threshold-crossing with a supralinear, exponential growth of the voltage [15] that resulted in a more realistic simulation of spike generation [32]. This passive exponential IF (PEx) model presented a rapid voltage increase when sufficiently depolarized (Fig 2C, right), while its subthreshold responses near the resting potential remained indistinguishable from the other two passive models (Fig 2A–2C).

Analogously to the passive models, we examined three different models from the group of active IF models (Figs 1A and 2D). The active leaky IF (ALk) model and the "active IF with spike current" (ASp) model had a fixed threshold for spike generation and either voltage reset (Fig 2D, left) or spike-mimicking current (Fig 2D, middle). After fitting the model parameters, the active exponential IF (AEx) model shared the subthreshold responses with the other active models (ALk/ASp) and suprathreshold responses to the PEx model (Fig 2D, right). The simulated excitatory and inhibitory postsynaptic potentials of all six models (Fig 2C and 2D) resembled physiological responses observed in previous in vitro studies of gerbil LSO [33]. In our fitting of parameters, we carefully considered the following points to enable systematic comparisons among these models (see also "Response criteria and parameter selection" in Materials and Methods). First, we adopted the same set of parameters that were used for our previous study [7], in which the PLk and ASp models were already examined. The sole exception was the refractory period that was changed from 1.6 ms to 2.0 ms to better fit our target criteria (see "Effects of refractory period" in Methods). Second, if a parameter was shared by more than one model, then we tried to use the same value for all models, at least within the category of passive or active models. Because of these constraints, the only remaining free parameters were for the spike generating currents of the exponential models (PEx and AEx), which were chosen to satisfy the response criteria described below.

### Common examination criteria

**Fig 3:**
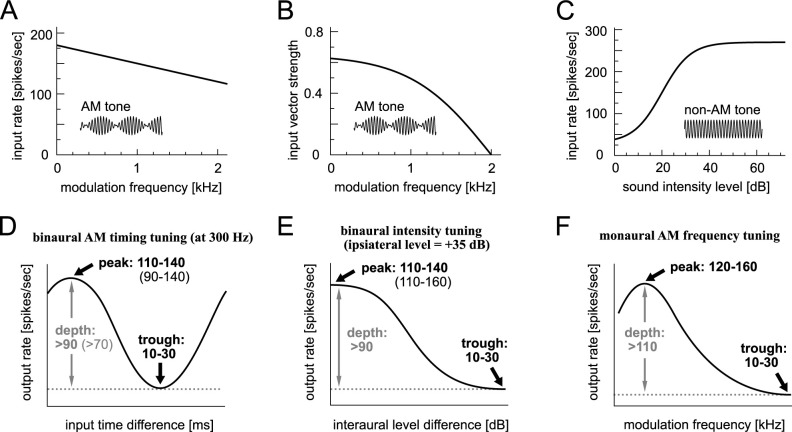
Settings of LSO model simulations. **A:** Modeled input rates driven by AM tones with varied modulation frequencies (Eq 8 in Materials and Methods). **B:** Modeled degree of input phase-locking (measured as vector strength) driven by AM tones with varied modulation frequencies (Eq 9). **C:** Modeled input spike rates driven by unmodulated tones with varied intensities (Eq 10). **D:** Targeted response of LSO models driven by binaural AM tones with varied input time (modulation phase) differences. **E:** Targeted response of LSO models driven by binaural unmodulated tone with varied ILD. **F:** Targeted response of LSO models driven by monaural AM tones with varied modulation frequency. In D and E, the "targeted" value ranges are shown in bold and the slightly larger ranges of "accepted" values that were applied to passive models are shown in parentheses.

Since the main goal of this study is to compare various IF models of an LSO neuron, we fixed the input stage and evaluated the output of each model neuron. We used the same set of synaptic parameter values as in our previous comparative modeling study [7] and applied it to all six IF models. In short, each LSO neuron model was assumed to receive 20 excitatory and 8 inhibitory inputs, with their spike rate and degree of phase-locking depending on the modulation frequency (Fig 3A and 3B). These phase-locked inputs underlie the monaural and binaural LSO responses [26]. To simulate ILD coding in LSO, we used a level-dependent, temporally uniform (non-phase-locked) input rate function (Fig 3C).

In our previous work [7], we set up common criteria for simulated monaural and binaural responses of modeled LSO neurons, based on available physiological data. We here adopted the same criteria to fit the parameters of the IF-type models. Namely, the maximum (peak) rate, the minimum (trough) rate, and their difference (depth) were examined for the time (Fig 3D) and level-difference tuning curves (Fig 3E). As shown previously for the ASp model [7] and as confirmed for other models in the next sections, the response rates of the active models were all within the "targeted" range (bold numbers in Fig 3D–3E). In the passive models that lack the KLVA conductance, however, the peak rates were generally higher for the time-difference tuning curve and lower for the level-difference tuning curve than in the active models. We thus loosened the criteria by using the "accepted" ranges for binaural tuning curves for the passive models (non-bold numbers in Fig 3D–3E). The model fitting was done at the modulation frequency of 300 Hz (for phase-difference tuning) and at the ipsilateral level of 35 dB (for level-difference tuning). Once fitting of the parameters was completed, the pre-set "targeted" values for the monaural AM frequency tuning curves (Fig 3F) were almost automatically satisfied by all models. In total, we set nine targeted ranges for monaural and binaural tuning curves and judged the physiological replicability of each model according to the number of ranges satisfied [7].

### Simulated traces and binaural tuning curves

**Fig 4:**
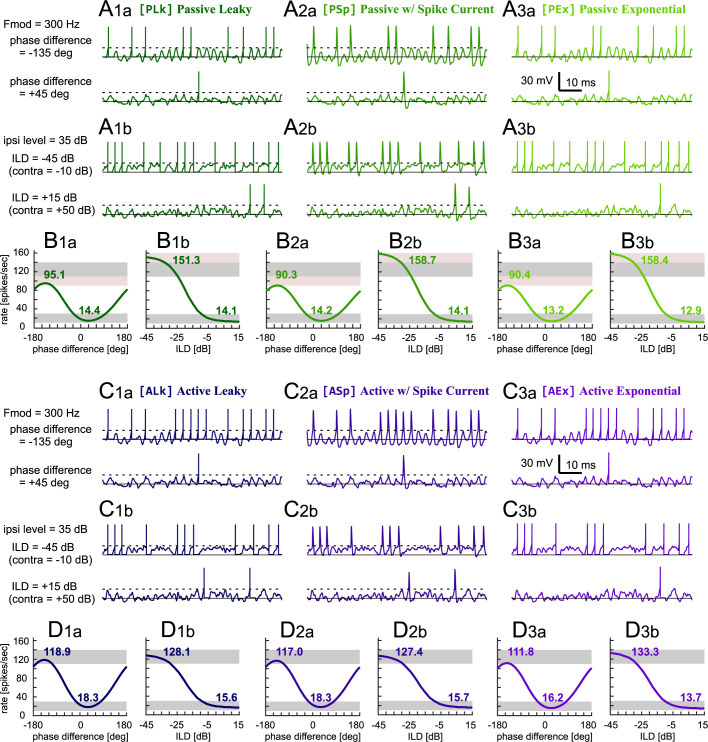
Simulated binaural tuning of LSO models. **A**_**1a**_**, A**_**2a**_**, A**_**3a**_: Simulated membrane potentials of the passive IF models driven by binaural AM tones with two different input phase differences. The phase differences of -135 deg and +45 deg corresponded approximately to the conditions in which the simulated spike rate becomes maximal and minimal, respectively. **A**_**1b**_**, A**_**2b**_**, A**_**3b**_: Simulated membrane potentials of the passive IF models driven by binaural unmodulated tones with two different ILDs, for which the contralateral inhibitory inputs were either very weak (-10 dB) or relatively intense (+50 dB). **B**_**1a**_**-B**_**3b**_: Output rates of the passive models in response to binaural AM tones with varied input phase differences (B_1a_, B_2a_, B_3a_) or to binaural unmodulated tones with varied ILDs (B_1b_, B_2b_, B_3b_). **C**_**1a**_**-C**_**3b**_: Same plots as in A_1a_-A_3b_, but for the three active models. **D**_**1a**_**-D**_**3a**_: Same plots as in B_1a_-B_3b_, but for the three active models. All six models were driven by identical inputs to facilitate comparisons. In A_1a_-A_3b_ and C_1a_-C_3b_, the horizontal solid and dotted lines mark the resting potentials and the spiking thresholds, respectively. In B_1a_-B_3b_ and D_1a_-D_3b_, the bold numbers show the peak and trough rates, and the light pink and gray rectangular shadings represent the accepted and targeted ranges, respectively (see Fig 3D–3E for their definitions). In the leaky models (A_1a_, A_1b_, C_1a_, C_1b_), vertical bars were manually added to show the timing of an output spike at each threshold crossing.

In this section, we describe the response characteristics of the six IF models driven by identical binaural inputs with time (phase) and level differences. The top two rows of Fig 4 (panels A_1a_, A_2a_, and A_3a_) show the simulated potential traces of the three passive IF models in response to stimuli phase-locked at 300 Hz. Regardless of their simulated spike shapes, their subthreshold responses and their spike timings generally resembled each other closely. This resemblance also held for level-difference tuning (Fig 4, panels A_1b_, A_2b_ and A_3b_), in which the input spike trains were simulated with homogeneous Poisson process and had therefore no special temporal structures. These results confirm that the passive models produced almost identical responses when they were driven by the same inputs. Small differences in their spike rates and timings, however, still originated from the different spike generation mechanisms. In contrast to the immediate voltage reset in the PLk model, the membrane potential of the PSp model did not fully recover from the simulated hyperpolarization even when the absolute refractory period was over at 2 ms after threshold crossing (Fig 2C). In the PEx model, exact spike timing was slightly (around 0.1 ms) delayed from the threshold crossing times of the other two models (Fig 2C), leading also to a delayed end of the absolute refractory period. Nevertheless, with the proper adjustment of the parameters, the phase and level tuning curves of the passive models were all within the pre-defined accepted ranges and were similar to each other (Fig 4B_1a_-4B_3b_).

The observations in the passive models were mostly mirrored in the active models (Fig 4C_1a_–4C_3b_). The three active-model subtypes presented very similar responses in phase-difference coding (Fig 4D_1a_–4D_3a_) as well as in level-difference coding (Fig 4D_1b_-4D_3b_). A notable discrepancy between the passive and active models, however, was the peak rate of the binaural tuning curves. The active models generated a larger number of spikes than the passive models for a favorable phase difference of -135 deg (compare Fig 4A_1a_–4A_3a_ and 4C_1a_–4C_3a_). In this case, hyperpolarization of the membrane caused by the phase-locked inhibitory inputs first led to the closing of KLVA channels, which in turn increased the membrane impedance (Fig 2A) and amplified the voltage response to the succeeding phase-locked excitatory inputs, resulting in an increased spiking probability. This KLVA-mediated enhancement of temporal signals is also depicted in the impedance plot (Fig 2B), and was seen in modeling [34] and experimental [35] studies of auditory coincidence detectors. In response to intense excitatory inputs (e.g., ILD = -45 dB), the opening of the KLVA channels in the active models reduced the membrane impedance (Fig 2A), thereby suppressing the depolarization of the membrane and resulting in a decreased spiking probability compared to the passive models (Fig 4A_1b_–4A_3b_ and 4C_1b_–4C_3b_). As the expression of KLVA conductance in LSO neurons is lower than in other prominent auditory coincidence detectors of MSO neurons or octopus cells [29,33,36], its simulated effects may not be as salient [37–41]. Nevertheless, our simulation results still suggest a considerable role of KLVA in suppressing the maximum response of level-difference tuning while amplifying the time-difference tuning. The combination of these effects enabled the active models to attain all targeted criteria for binaural tuning curves (Fig 3D–3E).

### Further monaural and binaural responses

**Fig 5:**
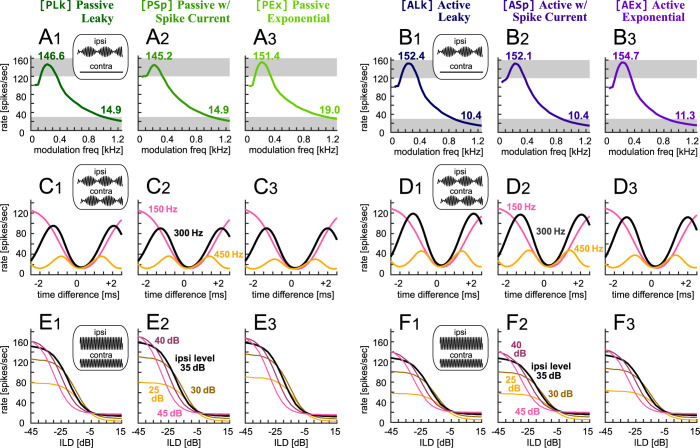
Simulated responses of LSO models to stimuli not used for model fitting. **A**_**1**_**-A**_**3**_ **and B**_**1**_**-B**_**3**_: Monaural AM-frequency-tuning curves of the passive (A_1_-A_3_) and active (B_1_-B_3_) IF models. Bold numbers show the peak rate and the rate at 1200 Hz. The gray shadings represent the targeted ranges (defined in Fig 3F). **C**_**1**_**-C**_**3**_ **and D**_**1**_**-D**_**3**_: Binaural AM-phase-tuning curves of the passive (C_1_-C_3_) and active (D_1_-D_3_) IF models at three modulation frequencies of 150, 300 and 450 Hz. **E**_**1**_**-E**_**3**_ **and F**_**1**_**-F**_**3**_: Binaural ILD-tuning curves of the passive (E_1_-E_3_) and active (F_1_-F_3_) IF models at five ipsilateral levels of 25–45 dB.

In the last section we confirmed that the simulated phase and level-difference tuning curves of all six models satisfy the physiology-based response criteria (Fig 3D–3E). Here we test the models with additional stimuli that were not used for parameter fitting. Simulated monaural AM frequency tuning curves of the passive (Fig 5A_1_–5A_3_) and active (Fig 5B_1_–5B_3_) models resembled each other, yielding curves within the pre-defined criteria (Fig 3F). The active models showed lower rates at modulation frequencies over 1 kHz, corresponding to a slightly steeper decrease of the membrane impedance with frequency (Fig 2B). Phase-tuning curves at a modulation frequency of 450 Hz were shallower in the passive models (Fig 5C_1_–5C_3_, orange) than in the active models (Fig 5D_1_–5D_3_, orange), because of the signal enhancement by the KLVA conductance discussed in the previous section. At 150 Hz modulation frequency, however, the difference between the passive and active models was small, with peak rates all within the range of 120–130 spikes/s (Fig 5C_1_–5D_3_, pink). This is due to the entrainment of spiking to the modulation frequency. Namely, the models elicited no more than one spike per modulation cycle, providing the active models with only marginal room for spike rate increase. The simulated level tuning curves showed a consistent compression of spike rates in the active models for all ipsilateral levels tested (Fig 5E_1_–5F_3_). To sum, the active models were more sensitive to temporal signals than the passive models and more compressive to high intensity signals. Within each active or passive model group, the diversity in the spike generation mechanisms led only to small differences in the tuning curves.

**Fig 6:**
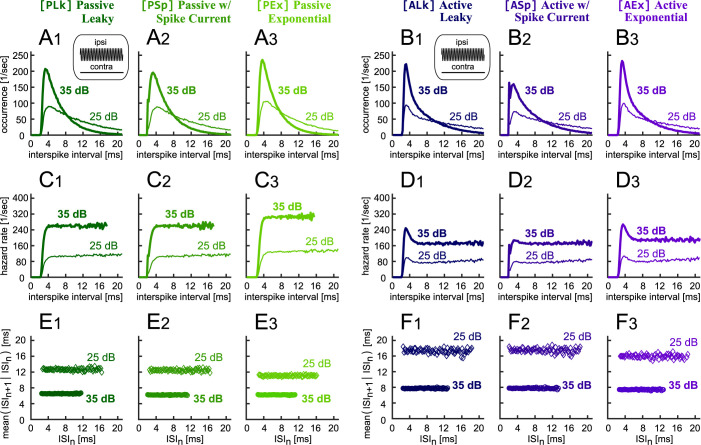
Simulated interspike-interval (ISI) statistics of LSO models to monaural tonal stimuli. **A**_**1**_**-A**_**3**_ **and B**_**1**_**-B**_**3**_: ISI histograms of the passive (A_1_-A_3_) and active (B_1_-B_3_) IF models at two intensities (25 and 35 dB SPL). **C**_**1**_**-C**_**3**_ **and D**_**1**_**-D**_**3**_: Recovery of hazard rate functions calculated from the corresponding ISI histograms. **E**_**1**_**-E**_**3**_ **and F**_**1**_**-F**_**3**_: Conditional mean of ISI with respect to the preceding ISI (see Materials and Methods for the definitions of these measures).

Temporal discharge patterns of LSO neurons were characterized with their interspike-interval (ISI) statistics. Earlier studies [42,43] found a variation of ISI histograms among LSO neurons in steady-state responses to monaural tonal stimulation. They also reported varied degrees of negative correlation of two succeeding ISIs. Zhou and Colburn [11] used an IF-type model equipped with afterhyperpolarization (AHP) conductance and showed that the observed cell-to-cell variability in ISI statistics can be simulated by adjusting the strength and time scale of AHP. In our simulations, both the passive (Fig 6A_1_–6A_3_) and active (Fig 6B_1_-6B_3_) IF models presented skewed ISI histograms, which were regarded as a signature of "fast chopping" neurons [42,43] and were associated with weak AHP [11]. The hazard rate (i.e., spike occurrence rate as a function of time after preceding spike) showed a quick recovery (Fig 6C_1_–6C_3_ and 6D_1_–6D_3_), corresponding to the response of model IF neurons with rapid release from AHP [11]. The simulated ISI histograms of the IF models with spike current (PSp and ASp) had a modest local peak at 2 ms (Fig 6A_2_ and 6B_2_), resembling some of those observed in vivo [43]. These models were able to fire immediately at the end of (absolute) refractory period, because, unlike the other four models, the membrane potential was not fixed to the reset potential. The active models had an increased spike probability at an ISI of 3–4 ms (Fig 6D_1_–6D_3_), which could be associated to the closing of KLVA channel during the refractory period. A similar hazard function was reported experimentally in cat LSO [42]. The conditional mean of ISIs showed a flat distribution for both passive (Fig 6E_1_–6E_3_) and active models (Fig 6F_1_–6F_3_), indicating a lack of serial dependence of two neighboring intervals, and resembling the empirical data from a small subset of LSO neurons [42,43]. In order to replicate a negative correlation between the intervals, an additional slow current that operates at a time scale of ~10 ms would have to be introduced in the model [11].

### Computational efficiency

In addition to physiological replicability, computational efficiency is another determining factor in selecting a model. The rightmost column of Table 1 lists the relative computational cost of each model in comparison to the coincidence counting (CoC) model (Fig 1A), which is the simplest LSO model among those we tested. The CoC model has no membrane potential but simply operates on the coincident arrival counts of excitatory and inhibitory inputs, allowing extremely fast computation [7,26]. Another reference for comparison, given in the last row of the model entries in Table 1, is the adjusted Wang-Colburn (AWC) model, a type of the Hodgkin-Huxley (HH) model [44] that simulates the temporal dynamics of several kinds of voltage-gated channels with differential equations. This model was the most complex and slowest in our collection of single-compartment LSO models [7].

**Table 1:** Summary of model output measures. Simulated spike rates that were within the targeted range are shown in **bold,** while those out of the targeted range but still within the accepted ranges are shown with regular fonts. Relative computation time is normalized to the average computation time of the coincidence counting model. The numbers in brackets with each model name indicate how many of the nine targeted ranges were attained. See Materials and Methods for the definition of each model and output measures.

Model	Monaural AM frequency coding			Binaural AM phase coding (at 300 Hz)			Binaural intensity coding (at ipsilateral level of 35 dB)			Relative computation time
	Peak (spikes/s)	Trough (spikes/s)	Depth (spikes/s)	Peak (spikes/s)	Trough (spikes/s)	Depth (spikes/s)	Peak (spikes/s)	Trough (spikes/s)	Depth (spikes/s)	
**[CoC]** Coincidence Counting (9/9)	**139.5**	**8.3**	**131.2**	**129.1**	**18.9**	**110.2**	**120.3**	**15.8**	**104.5**	1.0
**[PLk]** Passive Leaky IF (6/9)	**146.6**	**14.9**	**131.7**	95.1	**14.4**	80.7	151.3	**14.1**	**137.2**	2.7
**[PSp]** Passive IF with Spike Current (6/9)	**145.2**	**14.9**	**130.3**	90.3	**14.2**	76.1	158.7	**14.1**	**144.6**	3.0
**[PEx]** Passive Exponential IF (6/9)	**151.4**	**19.0**	**132.3**	90.4	**13.2**	77.2	158.4	**12.9**	**145.5**	5.9
**[ALk]** Active Leaky IF (9/9)	**152.4**	**10.4**	**142.0**	**118.9**	**18.3**	**100.6**	**128.1**	**15.6**	**112.5**	17.8
**[ASp]** Active IF with Spike Current (9/9)	**152.1**	**10.4**	**141.7**	**117.0**	**18.3**	**98.8**	**127.4**	**15.7**	**111.7**	18.4
**[AEx]** Active Exponential IF (9/9)	**154.7**	**11.3**	**143.4**	**111.8**	**16.2**	**95.6**	**133.3**	**13.7**	**119.6**	21.4
**[AWC]** Adjusted Wang-Colburn (9/9)	**158.4**	**23.9**	**134.5**	**117.9**	**24.8**	**93.1**	**114.2**	**22.1**	**92.1**	80.0
**Targeted** (and Accepted) **ranges**	**120–160**	**0–30**	**>110**	**110–140** (90–140)	**10–30**	**>90** (>70)	**110–140** (110–160)	**10–30**	**>90**	---

The calculated computational costs of the six IF models compared in this study were all between the two extreme cases of the CoC and the AWC models (Table 1). The passive models were 2–6 times slower than the CoC model and 13–30 times faster than the HH-type AWC model. The active models, whose simulation involved a step-by-step calculation of the voltage gated KLVA conductance, required considerably larger computational costs than the passive models. They were about 20 times slower than the CoC model, but still about 4 times faster than the HH-type model. In comparison to the switch from a passive model to an active model, the costs for modifying the spike generation mechanisms were relatively low. Introducing exponential spike generation (PEx/AEx models) added slightly higher computation time than introducing a spike-mimicking current (PSp/ASp models).

### Summary and concluding remarks

Having a selection of readily usable toolkits is beneficial in many fields of theoretical and experimental science, as they allow a user to select an approach suitable for the intended aims of the study. In this paper, we examined six different versions of IF models (Figs 1A and 2) and extended the list of available single-compartment LSO models [7] that were calibrated with common criteria. The simulated responses to monaural and binaural stimuli led us to the following conclusions. First, the active IF models were able to simulate physiological data more closely than the passive IF models, satisfying all the targeted response criteria (Figs 4 and 5; Table 1), but they required longer computation time. Second, the difference in spike generation mechanisms only marginally influenced the simulated physiological tunings and computational costs of both passive and active models (Table 1). Based on these results, in combination with our previous observations [7], we provide the following recommendations for selecting an LSO model:

- IF-type models, either passive or active, are suitable options for investigating the interaction between modeled synaptic inputs and the membrane responses, including the simulation of the developing or aging auditory system in which the synaptic strengths are altered separately from the membrane properties (e.g., [10,13]).
- Unless the computation time is a critical factor to consider (e.g., large-scale simulations involving thousands of neurons), an active model is superior to a passive model regarding the physiological plausibility and replicability, especially for temporally structured signals.
- If simulating spike shapes or replicating the spike-initiation mechanism is not a goal, the simplified threshold-crossing detection in the PLk and ALk models usually suffices.
- When a user has no strong preference for model selection, the active leaky (ALk) model will be a good starting point as a "medium-sized" LSO neuron model.
- To simulate sequential dependence of spike times that acts on the scale of over a few milliseconds, an additional mechanism (e.g., a slow current) needs to be introduced.
- If only the input-output relationship of an LSO neuron is considered, without simulating detailed synaptic inputs, a user can also choose the CoC model [26], which is even simpler than IF-type models. Such applications include the simulation of auditory-brainstem responses [45] and perception of binaural signals [46].

Limitations of our prior comparative modeling approach (see also Discussion in [7]) also apply to the present study. In our parameter fitting, we aimed to replicate "typical" LSO-like responses observed in various species. In physiological studies, however, activity of LSO neurons naturally shows variations even to the same stimuli. Therefore, fitting any model to a specific single neuron in a specific animal will require further tuning of the parameters. In addition, neurons in the LSO may have different membrane profiles along the tonotopic axis [36,47]. A passive model may be suitable for simulating purely low-pass neurons, while an active model with an increased KLVA conductance may be required for simulating neurons that have band-pass membrane impedances. Furthermore, physiologically distinct subpopulations of neurons exist even within each region of LSO [21,27]. In the present study, we simulated the activity of tonic spiking neurons, which continues to discharge in response to sustained sounds. Recent in vivo recordings, however, revealed a sharper binaural tuning of phasic (principal) neurons that responded only at the onset of acoustic stimulation, in comparison to tonic neurons [27,48]. A two-compartment Hodgkin-Huxley-type model was developed to simulate the synaptic integration and spike generation of phasic-spiking LSO neurons [48]. Creating an IF-version of phasic LSO model will be a subject of future investigations. Moreover, the modeled synaptic configurations (i.e., the number, strengths, time course, and fidelity of excitatory and inhibitory inputs) will also have to be adjusted, according to more detailed characterizations of synaptic connections to different LSO neurons in the future.

Fitting a complex HH-type model to a certain set of physiological data often requires substantial effort because of the large number of free parameters. IF-type models, in contrast, generally have far fewer parameters to fit and require a much shorter computation time for simulations. By separating and rearranging the ion-channel gating variables according to their time scales, an HH-type model can be reduced to a more easily tractable IF-type model, keeping its general spiking patterns intact (e.g., [49]). And with an appropriate modification, an IF-type model can replicate various types of spiking responses [17], providing further opportunities to simulate the physiological functions of LSO neurons efficiently and reliably with limited computational costs. We expect that the comparison of models in this study can be used as a guide for selecting a model for specific scientific questions and accompanying computational constraints.

## Materials and methods

### Integrate-and-fire models of LSO

We used the same configurations for the simulated synaptic inputs to drive our IF-type LSO models as in our previous study [7]. The names and the interrelations of the six IF-type models used in this study are shown by color in Fig 1A (passive models in green and active models in blue). All these models share the membrane equation of the form:

$$C(d/dt)V(t)=I_{all}=I_{synE}+I_{synI}+I_{L}+I_{K}+I_{exp}+I_{spike},$$ (1)

which describes how the membrane potential *V*(t) varies with the sum *I*_all_ of synaptic and membrane current. The excitatory and inhibitory synaptic currents (*I*_synE_ and *I*_synI_) are described in the next subsection. Both passive (PLk/PSp/PEx) and active (ALk/ASp/AEx) models have the leak current:

$$I_{L}=g_{L}\cdot (E_{L}‐V).$$ (2)

In addition, the active models have the low-voltage-activated potassium (KLVA) current:

$$I_{K}=g_{K}\cdot d(t)\cdot (E_{K}‐V).$$ (3)

Here, *d*(t) represents the fraction of open KLVA channels that varies with time according to the equation:

$$(d/dt)d(t)=\alpha_{d}(V)\cdot (1‐d(t))‐\beta_{d}(V)\cdot d(t).$$ (4)

In this equation, α_*d*_(*V*) = 0.5exp (+(*V*+50)/16) and β_*d*_(*V*) = 0.5exp(-(*V*+50)/16) are the activation and inactivation function of the KLVA channel in 1/ms, respectively [7]. In the passive models, the KLVA conductance is fixed to zero.

Both passive and active exponential IF models (PEx/AEx) have an exponential spike-generating current of the form [15]:

$$I_{exp}=g_{T}\cdot K_{T}\cdot exp((V‐V_{T})/K_{T}),$$ (5)

while in all other models this current is set to zero. In all models, a spike is counted when the membrane potential reaches the pre-set spike-detecting threshold *V*_th_. After crossing the threshold, the membrane potential of the leaky (PLk/ALk) and exponential (PEx/AEx) models is reset to the potential *V*_ref_ and held at that level for a refractory period of *T*_ref_. Threshold crossing in the models with spike currents (PSp/ASp) does not lead to an immediate voltage reset, but triggers a spike-mimicking current:

$$I_{spike}(t)=A_{1}\cdot exp(‐(t‐s)/\tau_{1})‐A_{2}\cdot exp(‐(t‐s)/\tau_{2}),$$ (6)

with *s* being the time of threshold crossing [7,30]. We used *A*_1_ = 24 (nA), *A*_2_ = 12 (nA), τ_1_ = 0.17 (ms), and τ_2_ = 0.37 (ms) for the passive IF model, and *A*_1_ = 24 (nA), *A*_2_ = 12 (nA), τ_1_ = 0.15 (ms), and τ_2_ = 0.30 (ms) for the active model. The resulting spike waveforms are shown in Fig 2C and 2D. The parameters of our IF models are summarized in Table 2. Justifications of the membrane parameters are provided in the "Response criteria and parameter selection" section.

**Table 2:** Parameters of the IF models. See text for the description of each parameter and Fig 1A for the abbreviations of the model names.

Parameter	PLk/PSp	PEx	ALk/ASp	AEx
Membrane capacitance C	24 pF	24 pF	24 pF	24 pF
Leak conductance g_L_	26.4 nS	26.4 nS	14.4 nS	14.4 nS
Leal reversal potential E_L_	-60 mV	-60 mV	-56 mV	-56 mV
KLVA conductance g_K_	---	---	21.6 nS	21.6 nS
KLVA reversal potential E_K_	---	---	-75 mV	-75 mV
Spike-generating conductance g_T_	---	26.4 nS	---	26.4 nS
Threshold factor *V*_T_	---	-46.6 mV	---	-47.3 mV
Slope factor *K*_T_	---	1.8 mV	---	1.8 mV
Spike-detection threshold *V*_th_	-45.1 mV	-10.0 mV	-45.8 mV	-10.0 mV
Reset potential *V*_ref_	-60 mV	-60 mV	-60 mV	-60 mV
Refractory period *T*_ref_	2.0 ms	2.0 ms	2.0 ms	2.0 ms

By eliminating the zero-current terms, the total current in the membrane equation can be written separately for each model as:

$$[PLk]\;I_{all}=I_{synE}+I_{synI}+I_{L},$$ (7A)

$$[PSp]\;I_{all}=I_{synE}+I_{synI}+I_{L}+I_{spike},$$ (7B)

$$[PEx]\;I_{all}=I_{synE}+I_{synI}+I_{L}+I_{exp},$$ (7C)

$$[ALk]\;I_{all}=I_{synE}+I_{synI}+I_{L}+I_{K},$$ (7D)

$$[ASp]\;I_{all}=I_{synE}+I_{synI}+I_{L}+I_{K}+I_{spike},$$ (7E)

$$[AEx]\;I_{all}=I_{synE}+I_{synI}+I_{L}+I_{K}+I_{exp}.$$ (7F)

In sum, all models have non-zero currents for the excitatory and inhibitory synaptic inputs and the membrane leak, while the other components additionally characterize the variation of IF models.

### Common synaptic inputs

Here, we briefly describe the fundamental settings of the common input model used to drive the IF models. For more detailed descriptions and justifications, see [7,26]. Based on available experimental data [33,50] and as in previous studies [7,26,44,45], each LSO neuron was assumed to receive synaptic inputs from 20 excitatory and 8 inhibitory afferents. Both excitatory and inhibitory input spike trains to the LSO model were simulated with an inhomogeneous Poisson process, whose intensity function was modeled with a von Mises distribution function that varies with time to mimic phase-locked spiking patterns of the input fibers [51,52].

For AM sound stimulation at a fixed level, the average intensity (arrival rate) function λ of the inhomogeneous Poisson process was assumed to depend on the modulation frequency:

$$\lambda (f_{m})=180‐0.03f_{m}(spikes/sec),$$ (8)

with *f*_m_ being the modulation frequency in Hz. The degree of phase-locking, measured with vector strength (VS: [52,53]), was assumed to depend on the modulation frequency as:

$$VS(f_{m})=0.65(1‐exp((f_{m}‐2000)/500))/(1+exp((f_{m}‐2000)/500)).$$ (9)

The specific numbers in these equations were determined based on empirical data (see [26] for further explanations). The curve shapes of these functions are shown in Fig 3A and 3B. To simulate phase-difference-coding of an LSO neuron, we varied the difference of the locking phases between the excitatory and inhibitory inputs.

To simulate ILD coding in response to non-modulated sounds, the average intensity function λ was assumed to be level-dependent:

λ(s)=30+240/(1+exp(‐(s‐20)/6.0)), (10)

with s being the sound level in decibels (dB). This monotonically increasing intensity function had a sigmoidal shape (Fig 1C), mimicking the rate-level curves of bushy cells and MNTB fibers (see references in [7]). For ILD-tuning curves, the spiking activity of input fibers was assumed to be non-phase-locked (i.e., VS = 0), leading to homogeneous Poisson spike trains. In the monaural AM stimulation, the inhibitory fibers were spontaneously active at a rate of 30 spikes per second with no phase-locking.

Each input spike was converted into an alpha function [54] to obtain a unitary synaptic conductance:

$$g_{syn}(t)=A_{syn}(t/\tau_{syn})exp(1‐t/\tau_{syn}).$$ (11)

All spikes from all input fibers were summed to obtain the total synaptic conductance g_tot_(t), which was then multiplied with the difference between the synaptic reversal potential and the membrane potential to obtain the synaptic input current:

$$I_{syn}=g_{tot}(t)\cdot (E_{syn}‐V(t)).$$ (12)

To drive our IF models, we calculated the excitatory *I*_synE_ and inhibitory *I*_synI_ synaptic inputs separately. As detailed in [7], we adopted *A*_syn_ = 3.5 (nS), τ_syn_ = 0.16 (ms), and E_syn_ = 0 (mV) for the excitatory synaptic input current *I*_synE_, and *A*_syn_ = 12 (nS), τ_syn_ = 0.32 (ms), and E_syn_ = -75 (mV) for the inhibitory synaptic input current *I*_synI_. Membrane responses to simulated synaptic inputs are shown in Fig 2C and 2D.

### Response criteria and parameter selection

In response to simulated binaural AM signals, the LSO models change their spike rates according to the time (phase) difference between the excitatory (ipsilateral) and inhibitory (contralateral) inputs (schematic graph in Fig 3D), which is termed the "phase-difference tuning". Driven by binaural non-phase-locked inputs, the models generally show a monotonic decrease of spike rate with the simulated ILD (schematic graph in Fig 3E), which is called the "level-difference tuning" here. To enable comparisons among the IF models of the present study and with other LSO models examined before, we re-used a set of criteria for these binaural tunings [7]. The active models were tuned to fit the "targeted" spiking rates for binaural phase-difference tuning at a modulation frequency of 300 Hz and for binaural level-difference tuning at an ipsilateral level of +35 dB (bold numbers in Fig 3D–3E and in Table 1, bottom row). The passive models, however, did not satisfy all these criteria (see "Simulated traces and binaural tuning curves" in Results and Discussion for the explanation of underlying mechanisms), and therefore the "accepted" spike rates were instead used for the peak (max) values of the binaural tuning curves, as well as for the depth (the difference between peak and trough) of the binaural phase-tuning curves (non-bold numbers in Fig 3D–3E and in Table 1, bottom row).

The selection and fitting of the model parameters were performed in the following manner.

- For all six models, the membrane capacitance C, reset voltage *V*_ref_, and refractory period *T*_ref_ were fixed to 24 pF, -60 mV, and 2 ms, respectively.
- For the remaining parameters of the PLk and ASp models, the same sets of parameters were adopted from our previous study [7]. Therefore, these models had no free parameters to adjust. The resulting membrane resistance at -60 mV was comparable between both models (about 38 MΩ; Fig 2A and 2B).
- The other two passive models (PSp and PEx) adopted the values for the leak conductance g_L_ and leak reversal potential E_L_ from the PLk model.
- The other two active models (ALk and AEx) adopted the values for the leak conductance g_L_, leak reversal potential E_L_, KLVA conductance g_K_, and KLVA reversal potential E_K_ from the ASp model.
- The PSp model used the same spike detection threshold as the PLk model. The spike-generating current of the PSp model was re-adjusted from the ASp model to fit a general spike shape of LSO [33] by accounting for the difference in their membrane impedances at depolarization (Fig 2A). At this step, no free parameter was left for the PSp model.
- The ALk models used the same spike detection threshold as the ASp model. At this step, no free parameter is left for the ALk model.
- For both exponential models (PEx and AEx), the spike generating conductance was fixed to 26.4 nS, which was the same as the leak conductance of the passive models.
- The remaining parameters of the exponential models were adjusted with grid search. A range from -50 to -40 mV (first with a coarse step of 0.5 ms, later with a finer step of 0.1 ms) for the threshold factor, from 1.2 mV to 4.0 mV (with a step of 0.2 mV) for the slope factor, and from -30 mV to 0 mV (with a step of 5 mV) for the spike detection threshold were used to find a parameter set to satisfy our binaural response criteria.

In our simulations, we calculated the average spike rates of the model neuron over 40 seconds for each parameter set. As in other models [7], more than one parameter combination attained the targeted ranges and produced similar results. Therefore, the values listed in Table 2 should be considered as one possible combination of the model parameters, but not as the "best fit".

### Effects of the refractory period

**Fig 7:**
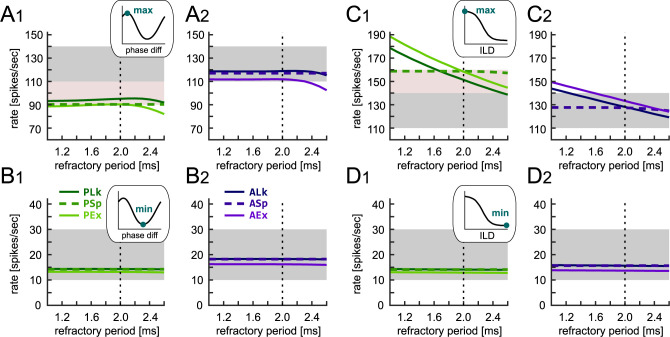
Effects of refractory period on binaural tuning curves. **A**_**1**_**-A**_**2**_ **and B**_**1**_**-B**_**2**_: Effect of the refractory period of the passive (A_1_ and B_1_) and active (A_2_ and B_2_) IF models on the peak (max: A_1_ and A_2_) and trough (min: B_1_ and B_2_) rates of binaural AM-phase-tuning curve at 300 Hz. **C**_**1**_**-C**_**2**_ **and D**_**1**_**-D**_**2**_: Effect of the refractory period of the passive (C_1_ and D_1_) and active (C_2_ and D_2_) IF models on the peak (max: C_1_ and C_2_) and trough (min: D_1_ and D_2_) rates of binaural ILD-tuning curve at the ipsilateral level of 35 dB. The vertical dotted line shows the refractory period of 2.0 ms used for our series of simulations. The targeted and accepted ranges are indicated by the gray and light pink rectangular shadings, respectively.

In our initial attempts, we tried to fit the model with a refractory period *T*_ref_ of 1.6 ms, which was adopted in our previous modeling studies [7,26]. The resulting peak spike rates of some IF models, however, consistently exceeded the designated (targeted or accepted) range in our extensive grid search (see above). Hence we extended the duration of the refractory period to 2.0 ms to adjust the spike rates. This adjustment was performed in the typical range of refractory periods for cat LSO neurons (1.1–2.8 ms [55]). We also investigated how a change in the refractory period altered the spike rates of each IF model in response to binaural inputs. The peak (Fig 7A_1_-7A_2_) and trough (Fig 7B_1_-7B_2_) rates of the phase-difference tuning curves were almost insensitive to the change in refractory period. The PLk/PEx/ALk/AEx models (solid lines) showed a decrease in peak rate for *T*_ref_ > 2.4 ms, with which the long refractory period starts to negatively impact the spike generation over a modulation period of 3.3 ms (corresponding to 300 Hz modulation). In the level-difference tuning curves, the increase in *T*_ref_ linearly reduced the peak rate of these four models (Fig 7C_1_-7C_2_), while the trough rates were not affected (Fig 7D_1_-7D_2_). Therefore, the parameter *T*_ref_ was used to finely tune the peak of level-difference tuning without affecting other response criteria. In the PSp and ASp models, however, change in refractory period had almost no influences, because the recovery after each spike was dominated by the spike-mimicking current that lasted longer than the refractory period (Fig 2C and 2D).

### Interspike-interval statistics

As an additional characterization of the IF models, we computed the spike interval statistics used in previous studies of LSO [42,43] (for theoretical accounts of the analyses, see, e.g., [56–58]). The LSO models were fed with the ipsilateral excitatory inputs that was used for binaural level-difference tuning curves; the contralateral inhibition was kept at the spontaneous level. ISI histograms were constructed with a time bin of *T*_bin_ = 0.2 ms. The ISI count in each bin (termed *N*_*i*_, with the subscript *i* indicating the *i*-th bin) was normalized with the product of the bin size and the total count of ISIs, to have a unit of 1/s. The hazard rate function *H*_*i*_ for each interval was calculated as: *H*_*i*_ = *N*_*i*_ / (*T*_bin_·*S*_*i*_), with the normalization factor *S*_i_ being the sum of all ISI counts for the *i*-th and higher bins [57]. We stopped the calculation of the hazard function when the value of *S*_*i*_ became smaller than 2% of the total count of ISIs.

For a second-order ISI statistic, we counted the number of occurrences for each pair of two neighboring intervals (ISI_n_, ISI_n+1_) in the simulated LSO spike trains. We used the same bin width *T*_bin_ as above. For each bin of ISI_n_, we took an average of ISI_n+1_ to have a conditional mean [56]. For a renewal process that has no memory of earlier spikes than the immediate prior, this conditional mean for ISI_n+1_ does not depend on the previous interval ISI_n_ [56,58]. We calculated the conditional means for intervals ISI_n_ that had more than 0.5% of the total ISI counts.

### Computation time and code availability

We used the forward Euler method for the numerical integration of the model equations with a time step 2 μs. We note, however, that a longer time step (up to around 10 μs) can generally be accepted for stable and accurate calculations of the IF models in the present study.

To evaluate the computational costs of each model, we calculated the total integration time of 40-second long traces and averaged it over 500 repetitions. In addition to the simulation of the six IF models, we also calculated the integration times of the coincidence counting (CoC) and the adjusted Wang-Colburn (AWC) models for further comparisons (see [7] for their definitions). To obtain relative computational costs, we normalized the integration time of each model by that of the CoC model, which was the fastest model introduced in our previous study [7].

Numerical algorithms were implemented in Matlab (version 2021a) and simulations were carried out on a desktop computer (Dell Precision T1700) with 64-bit Windows 7 Professional Operating System, Intel Xeon CPU E3-1270 v3 (4 core, 3.5 GHz) and a 16 GB memory. The model code is publicly available at https://github.com/pinkbox-models.

## References

[1] Boudreau JC, Tsuchitani C. Binaural interaction in the cat superior olive S segment. J Neurophysiol. 1968; 31: 442–454. doi: 10.1152/jn.1968.31.3.442 5687764

[2] Grothe B, Pecka M, McAlpine D. Mechanisms of sound localization in mammals. Physiol Rev. 2010; 90: 983–1012. doi: 10.1152/physrev.00026.2009 20664077

[3] Yin TCT, Smith PH, Joris PX. Neural mechanisms of binaural processing in the auditory brainstem, Compr Physiol. 2019; 9: 1503–1575. 10.1002/cphy.c180036. 31688966

[4] Colburn HS. Computational models of binaural processing. In: Hawkins HL et al. (eds). Auditory Computation. New York, Springer; 1996. pp 332–400. 10.1007/978-1-4612-4070-9_8.

[5] Jennings TR, Colburn HS. Models of the superior olivary complex. In: Meddis R et al. (eds). Computational models of the auditory system (Springer handbook of auditory research 35). New York, Springer; 2010. pp 65–96. 10.1007/978-1-4419-5934-8_4.

[6] Dietz M, Ashida G. Computational models of binaural processing. In: Litovsky R et al. (eds) Binaural hearing (Springer handbook of auditory research 73). New York, Springer; 2021. pp 281–315. 10.1007/978-3-030-57100-9_10.

[7] Ashida G, Tollin DJ, Kretzberg J. Physiological models of the lateral superior olive. PLoS Comput Biol. 2017; 13: e1005903. doi: 10.1371/journal.pcbi.1005903 29281618 PMC5744914

[8] Segev I. Single neurone models: oversimple, complex and reduced. Trends Neurosci. 1992: 15: 414–421. doi: 10.1016/0166-2236(92)90003-q 1281347

[9] Lapicque L. Recherches quantitatives sur l’excitation électrique des nerfs traitée comme une polarisation, J Physiol Pathol Gén 1907; 9: 620–635. Translation by Brunel N and van Rossum MCW. Quantitative investigations of electrical nerve excitation treated as polarization. Biol Cybern. 2007; 97: 341–349. 10.1007/s00422-007-0189-6. 18046573

[10] Fontaine B, Peremans H. Tuning bat LSO neurons to interaural intensity differences through spike-timing-dependent plasticity. Biol Cybern. 2007; 97: 261–267. doi: 10.1007/s00422-007-0178-9 17899163

[11] Zhou Y, Colburn HS. A modeling study of the effects of membrane afterhyperpolarization on spike interval statistics and on ILD encoding in the lateral superior olive. J Neurophysiol. 2010; 103: 2355–2371. doi: 10.1152/jn.00385.2009 20107123 PMC2867583

[12] Karcz A, Hennig MH, Robbins CA, Tempel BL, Rübsamen R, Kopp-Scheinpflug C. Low-voltage activated Kv1.1 subunits are crucial for the processing of sound source location in the lateral superior olive in mice. J Physiol. 2011; 589.5: 1143–1157. doi: 10.1113/jphysiol.2010.203331 21224222 PMC3060593

[13] Ashida G, Tollin DJ, Kretzberg J. Robustness of neuronal tuning to binaural sound localization cues against age-related loss of inhibitory synaptic inputs. PLoS Comput Biol 2021; 17: e1009130. doi: 10.1371/journal.pcbi.1009130 34242210 PMC8270189

[14] Latham PE, Richmond BJ, Nelson PG, Nirenberg S. Intrinsic dynamics in neuronal networks. I. theory. J Neurophysiol. 2000; 83: 808–827. doi: 10.1152/jn.2000.83.2.808 10669496

[15] Fourcaud-Trocmé N, Hansel D, van Vreeswijk C, Brunel N. How spike generation mechanisms determine the neuronal response to fluctuating inputs. J Neurosci. 2003; 23: 11628–11640. doi: 10.1523/JNEUROSCI.23-37-11628.2003 14684865 PMC6740955

[16] Jolivet R, Lewis TJ, Gerstner W. Generalized integrate-and-fire models of neuronal activity approximate spike trains of a detailed model to a high degree of accuracy. J Neurophysiol. 2004; 92: 959–976. doi: 10.1152/jn.00190.2004 15277599

[17] Naud R, Marcille N, Clopath C, Gerstner W. Firing patterns in the adaptive exponential integrate-and-fire model Biol Cybern. 2008; 99: 335–347. doi: 10.1007/s00422-008-0264-7 19011922 PMC2798047

[18] Joris PX, Yin TCT. Envelope coding in the lateral superior olive. III. Comparison with afferent pathways. J Neurophysiol. 1998; 79: 253–269. doi: 10.1152/jn.1998.79.1.253 9425196

[19] Tsai JJ, Koka K, Tollin DJ. Varying overall sound intensity to the two ears impacts interaural level difference discrimination thresholds by single neurons in the lateral superior olive. J Neurophysiol. 2010; 103: 875–886. doi: 10.1152/jn.00911.2009 20018829 PMC2822693

[20] Tollin DJ. The lateral superior olive: a functional role in sound source localization. Neuroscientist. 2003; 9: 127–143. doi: 10.1177/1073858403252228 12708617

[21] Friauf E, Krächan EG, Müller NIC. Lateral superior olive: organization, development, and plasticity. In: Kander K (ed) Oxford Handbook of the Auditory Brainstem. Oxford University Press, UK. 2019; pp 329–394. 10.1093/oxfordhb/9780190849061.013.10.

[22] Joris PX, Yin TCT. Envelope coding in the lateral superior olive. I. Sensitivity to interaural time differences. J Neurophysiol. 1995; 73: 1043–1062. doi: 10.1152/jn.1995.73.3.1043 7608754

[23] Irvine DRF, Park VN, McCormick L. Mechanisms underlying the sensitivity of neurons in the lateral superior olive to interaural intensity differences. J Neurophysiol. 2001; 86: 2647–2666. doi: 10.1152/jn.2001.86.6.2647 11731526

[24] Tollin DJ, Yin TCT. Interaural phase and level difference sensitivity in low-frequency neurons in the lateral superior olive. J Neurosci. 2005; 25: 10648–10657. doi: 10.1523/JNEUROSCI.1609-05.2005 16291937 PMC1449742

[25] Krips R, Furst M. Stochastic properties of auditory brainstem coincidence detectors in binaural perception. J Acoust Soc Am. 2009; 125: 1567–1583. doi: 10.1121/1.3068446 19275315

[26] Ashida G, Kretzberg J, Tollin DJ. Roles for coincidence detection in coding amplitude-modulated sounds. PLoS Comput Biol. 2016; 12: e100499. doi: 10.1371/journal.pcbi.1004997 27322612 PMC4920552

[27] Franken PT, Joris PX, Smith PH. Principal cells of the brainstem’s interaural sound level detector are temporal differentiators rather than integrators. eLife. 2018; 7: e33854. doi: 10.7554/eLife.33854 29901438 PMC6063729

[28] Johnston J, Forsythe ID, Kopp-Scheinpflug C. Going native: voltage-gated potassium channels controlling neuronal excitability. J Physiol.2010; 588.17: 3187–3200. doi: 10.1113/jphysiol.2010.191973 20519310 PMC2976014

[29] Golding NL, Oertel D. Synaptic integration in dendrites: exceptional need for speed. J Physiol. 2012; 590: 5563–5569. doi: 10.1113/jphysiol.2012.229328 22930273 PMC3528977

[30] Ashida G, Funabiki K, Kretzberg J. Minimal conductance-based model of auditory coincidence detector neurons. PLoS ONE. 2015; 10: e0122796. doi: 10.1371/journal.pone.0122796 25844803 PMC4386812

[31] Ashida G, Nogueira W. Spike-conducting integrate-and-fire model. eNeuro. 2018; 5: ENEURO.0112-18.2018. doi: 10.1523/ENEURO.0112-18.2018 30225348 PMC6140110

[32] Brette R. What is the most realistic single-compartment model of spike initiation? PLoS Comput Biol 2015; 11: e1004114. doi: 10.1371/journal.pcbi.1004114 25856629 PMC4391789

[33] Sanes DH. An in vitro analysis of sound localization mechanisms in the gerbil lateral superior olive. J Neurosci. 1990; 10: 3494–3506. doi: 10.1523/JNEUROSCI.10-11-03494.1990 2172478 PMC6570104

[34] Day ML, Doiron B, Rinzel J. Subthreshold K+ channel dynamics interact with stimulus spectrum to influence temporal coding in an auditory brain stem model. J Neurophysiol 2008; 99: 534–544, 2008. doi: 10.1152/jn.00326.2007 18057115 PMC3641780

[35] Beiderbeck B, Myoga MH, Müller NIC, Callan AR, Friauf E, Grothe B, Pecka M. Precisely timed inhibition facilitates action potential firing for spatial coding in the auditory brainstem. Nat Commun. 2018: 9: 1771. 10.1038/s41467-018-04210-y. PMC5932051 29720589

[36] Remme MWH, Donato R, Mikiel-Hunter J, Ballestero JA, Foster S, Rinzel J, McAlpine D. Subthreshold resonance properties contribute to the efficient coding of auditory spatial cues. Proc Natl Acad Sci USA. 2014; 111: E2339–E2348. doi: 10.1073/pnas.1316216111 24843153 PMC4050603

[37] Cai Y, Walsh EJ, McGee J. Mechanisms of onset responses in octopus cells of the cochlear nucleus: implications of a model. J Neurophysiol. 1997; 78: 872–883. doi: 10.1152/jn.1997.78.2.872 9307120

[38] Svirskis G, Kotak V, Sanes DH, Rinzel J. Sodium along with low-threshold potassium currents enhance coincidence detection of subthreshold noisy signals in MSO neurons. J Neurophysiol 2004; 91: 2465–2473. doi: 10.1152/jn.00717.2003 14749317 PMC3683536

[39] Mathews PJ, Jercog PE, Rinzel J, Scott LL, Golding NL. Control of submillisecond synaptic timing in binaural coincidence detectors by Kv1 channels. Nat Neurosci. 2010; 13: 601–609. doi: 10.1038/nn.2530 20364143 PMC3375691

[40] McGinley MJ, Liberman MC, Bal R, Oertel D. Generating synchrony from the asynchronous: compensation for cochlear traveling wave delays by the dendrites of individual brainstem neurons. J Neurosci, 2012; 32: 9301–9311. doi: 10.1523/JNEUROSCI.0272-12.2012 22764237 PMC3417346

[41] Spencer MJ, Grayden DB, Bruce IC, Meffin H, Burkitt AN. An investigation of dendritic delay in octopus cells of the mammalian cochlear nucleus. Front Comput Neurosci. 2012; 6:83. doi: 10.3389/fncom.2012.00083 23125831 PMC3486622

[42] Tsuchitani C, Johnson DH. The effects of ipsilateral tone burst stimulus level on the patterns of cat lateral superior olivary units. J Acoust Soc Am. 1985; 77:1484–1496. 10.1121/1.392043. 2985673

[43] Johnson DH, Tsuchitani C, Linebarger DA, Johnson MJ. Application of a point process model to responses of cat lateral superior olive units to ipsilateral tones. Hear Res. 1986; 21: 135–169. doi: 10.1016/0378-5955(86)90035-3 3700253

[44] Wang L, Colburn HS. A modeling study of the responses of the lateral superior olive to ipsilateral sinusoidally amplitude-modulated Tones. J Assoc Res Otolaryngol. 2012; 13: 249–267. doi: 10.1007/s10162-011-0300-5 22160752 PMC3298618

[45] Brown AD, Anbuhl KL, Gilmer JI, Tollin DJ. Between-ear sound frequency disparity modulates a brain stem biomarker of binaural hearing. J Neurophysiol. 2019; 122: 1110–1122. doi: 10.1152/jn.00057.2019 31314646 PMC6766741

[46] Klug J, Schmors L, Ashida G, Dietz M. Neural rate difference model can account for lateralization of high-frequency stimuli. J Acoust Soc Am. 2020; 148: 678–691. doi: 10.1121/10.0001602 32873019

[47] Barnes-Davies M, Barker MC, Osmanu F, Forsythe ID. Kv1 currents mediate a gradient of principal neuron excitability across the tonotopic axis in the rat superior olive. Eur J Neurosci. 2004; 19: 325–333. 10.1111/j.0953-816X.2003.03133.x. 14725627

[48] Franken PT, Bondy BJ, Haimes DB, Goldwyn JH, Goding NL, Smith PH, Joris PX. Glycinergic axonal inhibition subserves acute sensitivity to sudden increases in sound intensity. eLife. 2021; 10: e62183. 10.7554/eLife.62183. 34121662 PMC8238506

[49] Meng X, Lu Q, Rinzel J. Control of firing patterns by two transient potassium currents: leading spike, latency, bistability. J Comput Neurosci. 2011; 31:117–136. doi: 10.1007/s10827-010-0297-5 21181249 PMC3630519

[50] Gjoni E, Aguet C, Sahlender DA, Knott G, Schneggenburger R. Ultrastructural basis of strong unitary inhibition in a binaural neuron. J Physiol. 2018; 596.20: 4969–4982. 10.1113/JP276015. PMC6187040 30054922

[51] Ashida G, Funabiki K, Carr CE. Theoretical foundations of the sound analogue membrane potential that underlies coincidence detection in the barn owl. Front Comput Neurosci. 2013; 7: 151. 10.3389/fncom.2013.00151. 24265616 PMC3821005

[52] Kessler D, Carr CE, Kretzberg J, Ashida G. Theoretical relationship between two measures of spike synchrony: correlation index and vector strength. Front Neurosci. 2021; 15: 761826. doi: 10.3389/fnins.2021.761826 34987357 PMC8721039

[53] Goldberg JM, Brown PB. Response of binaural neurons of dog superior olivary complex to dichotic tonal stimuli: some physiological mechanisms of sound localization. J Neurophysiol. 1969; 32: 613–636. doi: 10.1152/jn.1969.32.4.613 5810617

[54] Rotter S, Diesmann M. Exact digital simulation of time-invariant linear systems with applications to neuronal modeling. Biol Cybern. 1999; 81: 381–402. doi: 10.1007/s004220050570 10592015

[55] Tsuchitani C. The inhibition of cat lateral superior olive unit excitatory responses to binaural tone bursts. II. The sustained discharges. J Neurophysiol. 1988; 59: 184–211. doi: 10.1152/jn.1988.59.1.184 3343600

[56] Rodieck RW, Kiang NYS, Gerstein GL. Some quantitative methods for the study of spontaneous activity of single neurons. Biophys J. 1962; 2:351–368. doi: 10.1016/s0006-3495(62)86860-x 14493108 PMC1366397

[57] Gray PR. Conditional probability analysis of the spike activity of single neurons. Biophys J. 1967; 7: 759–777. 10.1016/S0006-3495(67)86621-9. 19210997 PMC1368191

[58] Johnson DH. Point process models of single-neuron discharges. J Comput Neurosci. 1996; 3: 275–299. doi: 10.1007/BF00161089 9001973

